# Differential expression of major histocompatibility complex class I in subtypes of breast cancer is associated with estrogen receptor and interferon signaling

**DOI:** 10.18632/oncotarget.8798

**Published:** 2016-04-18

**Authors:** Hee Jin Lee, In Hye Song, In Ah Park, Sun-Hee Heo, Young-Ae Kim, Jin-Hee Ahn, Gyungyub Gong

**Affiliations:** ^1^ Department of Pathology, University of Ulsan College of Medicine, Asan Medical Center, Seoul, Korea; ^2^ Department of Oncology, University of Ulsan College of Medicine, Asan Medical Center, Seoul, Korea

**Keywords:** breast carcinoma, tumor-infiltrating lymphocytes, major histocompatibility complex I, human leukocyte antigen, Pathology Section

## Abstract

Tumor-infiltrating lymphocytes (TILs) in triple-negative breast cancer (TNBC) have a strong prognostic and predictive significance. However, the mechanism of TIL influx in TNBC is unclear. Expression of major histocompatibility complex class I (MHC I) on the tumor cell is essential for the effective killing of tumor by cytotoxic TILs. In our current study, human leukocyte antigen (HLA) expression was inversely correlated with estrogen receptor (ER) expression in normal and cancerous breast tissue and positively correlated with TILs in breast cancer. The ER score was inversely correlated with TILs in breast cancer. *HLA-A* and *CD8B* gene expression was negatively correlated with *ESR1* and positively correlated with interferon-associated gene expression in The Cancer Genome Atlas (TCGA) data. Negative correlation between *ESR1* and *HLA* and positive correlation between interferon-associated and *HLA* gene expression were also confirmed in Cancer Cell Line Encyclopedia (CCLE) data. Taken together, our data suggest that a lower expression of HLA in luminal-type tumors might be associated with low level of TILs in those tumors. Further investigation of the mechanism of higher HLA expression and TIL influx in TNBC may help to boost the host immune response.

## INTRODUCTION

The importance of tumor-infiltrating lymphocytes (TILs) in breast cancer has been consistently documented [1–7]. TILs have a strong prognostic and predictive significance, particularly in triple-negative breast cancer (TNBC). Cytotoxic CD8^+^ TILs are only activated by T cell receptor-recognition of a specific peptide, which is mostly generated from endogenous proteins, presented by a major histocompatibility complex class I (MHC I) on the surface of tumor cells [8]. The recognition of these peptides by cytotoxic CD8^+^ TILs triggers a series of events that can result in tumor cell lysis. Better understanding of TILs and related features could facilitate the development of efficient immunotherapeutic approaches in breast cancer.

MHC I proteins are membrane proteins expressed on almost all nucleated cells and encoded by human leukocyte antigen (*HLA*)-*A*, -*B*, and -*C* genes [9]. Expression of *HLA*s varies from tissue to tissue and is largely stimulated by interferon (IFN) signaling [10, 11]. Downregulation of *HLA*s is frequently seen in tumors and is reported to be correlated with disease progression [10]. Aberrant HLA expression in tumor cells might be caused by alteration of *HLA* gene transcription, translation of *HLA* mRNA, or post-translational modification. Torigoe et al. [12] established a monoclonal anti-pan HLA class I antibody suitable for immunostaining of formalin-fixed tissue and found a high rate (85%, 35 out of 41 cases) of HLA downregulation in breast cancer compared with other malignancies (20%-42%). Since HLA expression on tumor cells is important for the function of TILs, downregulation of HLA might compromise the effective immune response in patients with breast cancer. Moreover, increased IFN signaling in cancer cells and their association with good response to anthracycline-based chemotherapy have been recently reported in breast cancer [13]. However, HLA expression, the level of IFN signaling activation, and their relationship in normal breast tissue and each subtype of breast cancer have not been extensively studied.

In our previous study, we reported that HLA-ABC and HLA-A expressions were positively correlated with TILs in HER2^+^ tumors that had been treated with adjuvant trastuzumab (Spearman correlation: rho = 0.246, *P* < 0.001 for HLA-ABC expression and TILs; rho = 0.249, *P* < 0.001 for HLA-A expression and TILs) [14]. However, HLA expression was not associated with the *HER2* gene amplification or HER2 overexpression, which may suggest that HER2 itself is not the factor that influences the level of TILs. HER2^+^ breast cancer and TNBC are well known to be associated with increased cancer cell proliferation and genomic instability but interestingly, TIL levels were found to be higher in both HER2^+^ breast cancer and TNBC than in ER^+^/HER2^−^ tumors [1]. We therefore hypothesized that genomic instability would produce more mutations, some of which are presented on tumor cells by HLA proteins, and induce a potent anti-tumor immune response. Consequently, an increased immune reaction would produce high levels of interferon-gamma (IFNγ), which can induce transcription of the *HLA* gene [10]. However, the relationships between the mutation rate and degree of TIL or HLA expression have not been studied in each type of breast cancer.

In our current study, we evaluated TILs and expression of HLA-ABC in two cohorts of breast cancer and HLA-ABC expression in normal breast tissue. The relationship among expression of *ESR1*, *HLA*s, and IFN-associated molecules was analyzed from The Cancer Genome Atlas (TCGA) and Cancer Cell Line Encyclopedia (CCLE) data. We also investigated correlation between *HLA* gene expression and mutation rate from TCGA data.

## RESULTS

### TILs and expression of HLA class I in breast cancer samples

**Figure 1:**
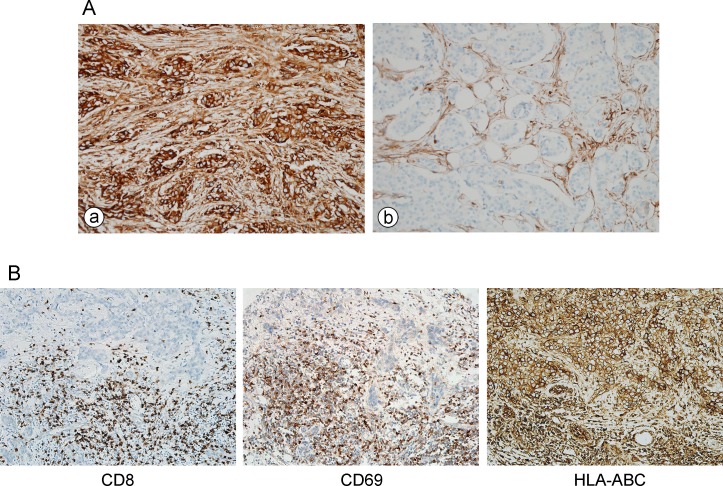
**A.** Representative figures of HLA-ABC expression in breast cancer. (a) Tumor and stromal cells strongly positive for HLA-ABC. (b) Tumor cells negative for HLA-ABC. **B.** CD8 and CD69 expressing cells and HLA-ABC expression in tumor cells of a case in validation TNBC cohort.

To explore the expression of HLA and its relationship with TIL in each subtype of breast cancer, we analyzed 688 consecutive breast cancer cohort (Table 1). The histologic grade and TIL levels were higher in TNBC and hormone receptor negative (HR^−^)/HER2^+^ tumors. While 22% of HR^+^/HER2^−^ tumors showed strong HLA-ABC expression in tumor cells, more than half of TNBCs were strongly positive for HLA-ABC by immunohistochemistry (Figure 1A). Lymphocytes were strongly positive for HLA-ABC in all subtypes and stromal cells in adjacent stroma of TNBC and HR^−^/HER2^+^ tumors showed stronger HLA-ABC expression than those of HR^+^ tumors. In all tumors, the ER Allred score was inversely correlated with the HLA-ABC immunoreactive score (rho = −0.177, *P* < 0.001) and TIL percentage (rho = −0.378, *P* < 0.001). HLA-ABC expression was significantly correlated with TIL level (rho = 0.442, *P* < 0.001).

**Table 1:** Comparison of pathologic variables according to breast cancer subtype in the first consecutively resected cohort

Variables	HR^+^/HER2^−^	HR^+^/HER2^−^	HR^−^/HER2^+^	TNBC	*P*
Histologic type					0.016
Invasive carcinoma of no special type	331 (88.7)	43 (97.7)	75 (92.6)	174 (91.6)	
Carcinoma with medullary features	0 (0.0)	0 (0.0)	0 (0.0)	4 (2.1)	
Carcinoma with mucinous features	3 (0.8)	0 (0.0)	0 (0.0)	0 (0.0)	
Invasive micropapillary carcinoma	12 (3.2)	1 (2.3)	5 (6.2)	5 (2.6)	
Mucinous carcinoma	10 (2.7)	0 (0.0)	1 (1.2)	0 (0.0)	
Metaplastic carcinoma	1 (0.3)	0 (0.0)	0 (0.0)	4 (2.1)	
Invasive lobular carcinoma	14 (3.8)	0 (0.0)	0 (0.0)	3 (1.6)	
Tubular carcinoma	2 (0.5)	0 (0.0)	0 (0.0)	0 (0.0)	
Histologic grade					<0.001
1	82 (22.0)	4 (9.1)	1 (1.2)	4 (2.1)	
2	217 (58.2)	25 (56.8)	34 (42.0)	51 (26.8)	
3	74 (19.8)	15 (34.1)	46 (56.8)	135 (71.1)	
pT					<0.001
1	151 (40.5)	15 (34.1)	19 (23.5)	51 (26.8)	
2	189 (50.7)	23 (52.3)	49 (60.5)	110 (57.9)	
3	28 (7.5)	5 (11.4)	12 (14.8)	20 (10.5)	
4	5 (1.3)	1 (2.3)	1 (1.2)	9 (4.7)	
Lymph node metastasis					0.731
Negative	176 (48.1)	15 (34.1)	34 (42.0)	94 (50.8)	
Positive	190 (51.9)	29 (65.9)	47 (58.0)	91 (49.2)	
Lymphovascular invasion					0.064
Negative	248 (66.5)	27 (64.3)	48 (59.3)	145 (76.3)	
Positive	125 (33.5)	15 (35.7)	33 (40.7)	45 (23.7)	
pTNM stage					0.044
I	100 (26.8)	7 (15.9)	14 (17.3)	38 (20.0)	
II	173 (46.4)	22 (50.0)	35 (43.2)	96 (50.5)	
III	99 (26.5)	15 (34.1)	32 (39.5)	54 (28.4)	
IV	1 (0.3)	0 (0.0)	0 (0.0)	2 (1.1)	
Hormone therapy					<0.001
Negative	74 (19.8)	5 (11.4)	36 (44.4)	114 (60.0)	
Positive	299 (80.2)	39 (88.6)	45 (55.6)	76 (40.0)	
Radiotherapy					0.254
Negative	283 (75.9)	27 (61.4)	60 (74.1)	135 (71.1)	
Positive	90 (24.1)	17 (38.6)	21 (25.9)	55 (28.9)	
Chemotherapy					<0.001
None	171 (45.8)	10 (22.7)	20 (24.7)	38 (20.0)	
Unknown regimen	8 (2.1)	0 (0.0)	0 (0.0)	6 (3.2)	
Anthracycline-based	36 (9.7)	12 (27.3)	16 (19.8)	26 (13.7)	
Methotrexate-based	158 (42.4)	22 (50.0)	45 (55.6)	120 (63.2)	
Tumor-infiltrating lymphocytes					<0.001
≤10%	283 (75.8)	27 (61.4)	31 (38.3)	62 (32.6)	
20–30%	57 (15.3)	12 (27.3)	25 (30.9)	50 (26.3)	
40%–60%	19 (5.1)	5 (11.3)	12 (14.8)	34 (17.9)	
>60%	14 (3.8)	0 (0.0)	13 (16.0)	44 (23.2)	
HLA-ABC expression in tumor cells					<0.001
Negative	213 (58.7)	16 (37.2)	38 (47.5)	64 (34.4)	
Weakly positive	70 (19.3)	15 (34.9)	17 (21.3)	26 (14.0)	
Strongly positive	80 (22.0)	12 (27.9)	25 (31.3)	96 (51.6)	
HLA-ABC intensity in stromal cells					<0.001
1	53 (14.5)	1 (2.3)	1 (1.2)	8 (4.3)	
2	221 (60.2)	27 (62.8)	35 (43.2)	70 (37.2)	
3	93 (25.3)	15 (34.9)	45 (55.6)	110 (58.5)	

HR, hormone receptor; TNBC, triple-negative breast cancer.

Since TILs are abundant (28.7 ± 27.7% in TNBC; 15.9 ± 22.2% in all tumors) and strong HLA-ABC expression is most frequent in TNBC, we analyzed the correlation between TILs and HLA-ABC expression and their prognostic significance in TNBCs included in our consecutive breast cancer series and validated the relationship in a separate cohort with a large number of TNBCs. In TNBCs within the first consecutive breast cancer cohort, a strong HLA-ABC expression was significantly correlated with a higher histologic grade, absence of lymphovascular invasion, basal type, higher TIL level, and stronger HLA-ABC expression in stromal cells (Table 2). In the validation TNBC cohort, a strong HLA-ABC expression in tumor cells was also significantly correlated with a higher histologic grade, basal type, higher TIL level, and stronger HLA-ABC expression in stromal cells (Supplementary Table S1). We also examined the numbers of CD8^+^ T cells and CD69^+^ activated immune cells in this validation TNBC cohort, and strong positive correlations were found between the numbers of CD8^+^ or CD69^+^ cells and the HLA-ABC expression in tumor cells as well as the TIL levels assessed on the H&E stained slides. (Figure 1B and Table 3).

**Table 2:** Comparison of pathologic factors in TNBCs within the consecutive breast cancer cohort according to HLAABC expression in tumor cells

Variables	HLA-ABC expression			
	Negative	Weakly positive	Strongly positive	*P*
Histology				0.008
Invasive carcinoma of no special type	62 (96.9)	20 (77.0)	89 (92.7)	
Carcinoma with medullary features	0 (0.0)	1 (3.8)	3 (3.1)	
Invasive micropapillary carcinoma	2 (3.1)	1 (3.8)	2 (2.1)	
Metaplastic carcinoma	0 (0.0)	2 (7.7)	2 (2.1)	
Invasive lobular carcinoma	0 (0.0)	2 (7.7)	0 (0.0)	
Histologic grade				0.001
1	2 (3.1)	1 (3.8)	0 (0.0)	
2	24 (37.5)	9 (34.6)	17 (17.7)	
3	38 (59.4)	16 (61.5)	79 (82.3)	
pT				0.140
1	16 (25.0)	7 (26.9)	27 (28.1)	
2	33 (51.6)	17 (65.4)	58 (60.4)	
3	10 (15.6)	2 (7.7)	7 (7.3)	
4	5 (7.8)	0 (0.0)	4 (4.2)	
Lymphovascular invasion				0.010
Negative	42 (65.6)	20 (76.9)	80 (83.3)	
Positive	22 (34.4)	6 (23.1)	16 (16.7)	
Lymph node metastasis				0.124
Negative	26 (42.6)	13 (50.0)	52 (55.3)	
Positive	35 (57.4)	13 (50.0)	42 (44.7)	
Adjuvant systemic therapy				0.165
None	16 (25.0)	5 (19.2)	16 (16.7)	
Unknown regimen	1 (1.6)	0 (0.0)	5 (5.2)	
Anthracycline-based	12 (18.7)	5 (19.2)	8 (8.3)	
Methotrexate-based	35 (54.7)	16 (61.6)	67 (69.8)	
Radiation therapy				0.072
Negative	42 (65.6)	16 (61.5)	75 (78.1)	
Positive	22 (34.4)	10 (38.5)	21 (21.9)	
Hormone therapy				0.559
Negative	37 (57.8)	16 (61.5)	60 (62.5)	
Positive	27 (42.2)	10 (38.5)	36 (37.5)	
Basal type				0.014
Negative	40 (62.5)	18 (69.2)	42 (43.8)	
Positive	24 (37.5)	8 (30.8)	54 (56.3)	
Tumor-infiltrating lymphocytes				<0.001
≤10%	32 (50.0)	14 (53.8)	16 (16.7)	
20–30%	21 (32.8)	5 (19.2)	21 (21.9)	
40%–60%	7 (10.9)	3 (11.5)	24 (25.0)	
>60%	4 (6.3)	4 (15.4)	35 (36.5)	
HLA-ABC intensity in stromal cells				<0.001
1	6 (9.4)	1 (3.8)	1 (1.0)	
2	38 (59.4)	13 (50.0)	17 (17.7)	
3	20 (31.3)	12 (46.2)	78 (81.3)	

**Table 3:** Correlations among variables on lymphocytes and HLA-ABC expression in the validation TNBC cohort (correlation coefficient rho and *P* value)

	Number of CD8^+^ cells	Number of CD69^+^ cells	HLA-ABC expression
Tumor infiltrating lymphocyte on H&E staining	0.627 (<0.001)	0.674 (<0.001)	0.420 (<0.001)
Number of CD8^+^ cells		0.869 (<0.001)	0.467 (<0.001)
Number of CD69^+^ cells			0.571 (<0.001)

**Figure 2:**
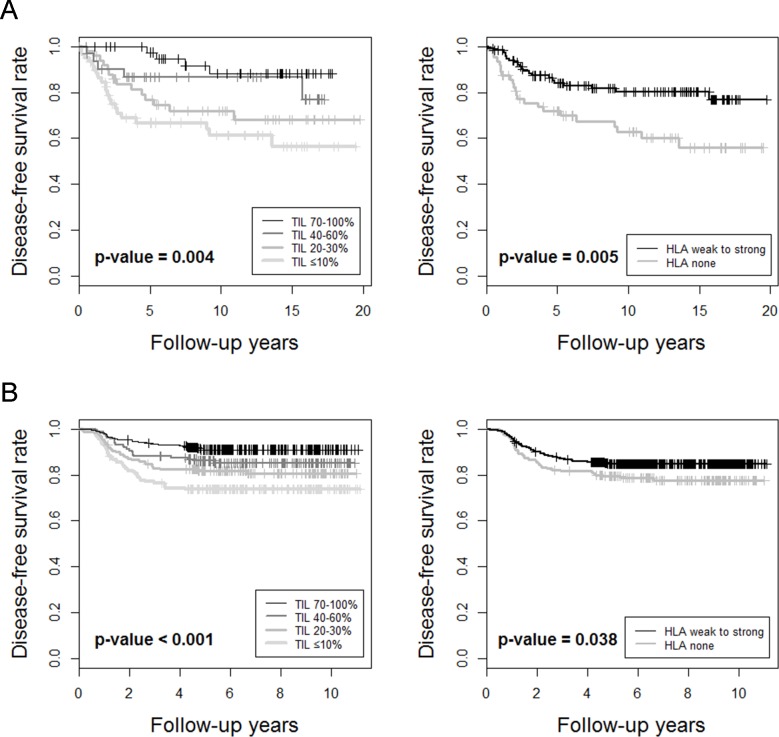
**Kaplan-Meier survival curves for A.** TNBCs in the consecutive breast cancer cohort and **B.** validation TNBC cohort, in terms of Tumor-infiltrating lymphocyte levels and HLA-ABC expression levels in tumor cells.

The prognostic significance of TILs and other clinicopathologic variables in TNBCs was analyzed. In TNBCs within the consecutive breast cancer cohort, higher histologic grade, lower pT stage, absence of lymph node metastasis, higher TIL level, and presence of HLA-ABC expression were associated with better disease-free survival (Table 4 and Figure 2A). In multivariate analysis, only lymph node metastasis and TIL level were independent prognostic factors for disease-free survival. In the validation TNBC cohort, lower pT stage, absence of lymph node metastasis, higher TIL level, and presence of HLA-ABC expression were also associated with better outcome (Supplementary Table S2 and Figure 2B). Again, lymph node metastasis and TIL level were independent prognostic factors for disease-free survival in this validation group.

**Table 4:** Survival analyses of clinicopathologic variables that affect clinical outcomes of TNBC cases in the consecutive breast cancer cohort

Variables	Univariate			Multivariate		
	Hazard ratio	95% CI	*P* value	Hazard ratio	95% CI	*P* value
Age: ≥50 years *vs*. <50	0.952	0.515–1.763	0.876	1.198	0.615-2.336	0.595
Histologic grade: 3 *vs*. 1/2	0.713	0.585–0.868	0.001	0.904	0.720-1.134	0.381
pT: 3/4 *vs*. 1/2	3.316	1.732–6.345	<0.001	1.632	0.794-3.356	0.183
Lymph node metastasis: positive *vs*. negative	7.729	3.247–18.395	<0.001	5.877	2.396-14.413	<0.001
TILs (per 10%)	0.975	0.960–0.989	0.001	0.980	0.963-0.997	0.023
HLA-ABC expression: strongly/weakly positive *vs*. negative	0.661	0.492–0.889	0.005	0.886	0.626-1.255	0.496

CI, confidence interval; TIL, tumor infiltrating lymphocyte.

### HLA-ABC expression in non-neoplastic breast tissue

**Figure 3:**
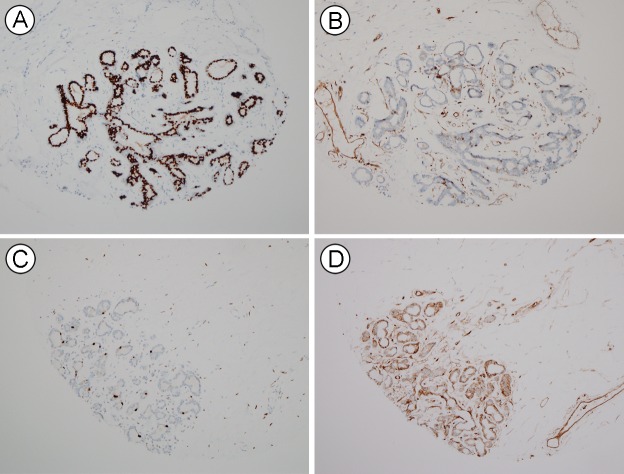
HLA-ABC and ER expression in normal breast luminal cells **A.**, **B.** A lobule with high ER (A) and low HLA-ABC (B) expression. **C.**, **D.** Another lobule with low ER (C) and high HLA-ABC (D) expression.

Because HLA-ABC expression was lowest in HR^+^/HER2^−^ tumors in our consecutive breast cancer samples, we hypothesized that the expression level of HLA-ABC might be associated with HR status. We examined ER, cytokeratin 5 (CK5), and HLA-ABC expression in tissue from reduction mammoplasty and mammary hamartoma cases. The ER Allred scores varied in each case (a score of 5 in 4 cases, 6 in 9 cases, 7 in 10 cases, and 8 in 14 cases). Compared with the expression intensity of HLA-ABC in breast cancer, normal luminal cells generally weakly expressed HLA-ABC (Figure 3). The immunoreactive score of HLA-ABC expression was inversely correlated with the ER Allred score (rho = −0.339, *P* = 0.04) in normal luminal cells between patients. CK5 expression was not associated with ER (rho = 0.029, *P* = 0.864) or HLA-ABC (rho = 0.005, *P* = 0.978) expression. We also examined ER and HLA-ABC expression in normal breast tissue of 45 cancer patients, and also found a significantly negative correlation (rho = −0.307, *P* = 0.036).

### Analysis of TCGA data

**Figure 4:**
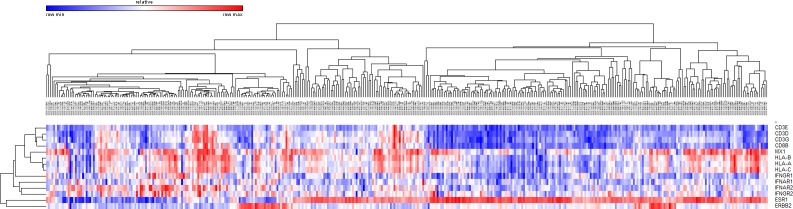
Unsupervised hierarchical clustering of breast cancers in the TCGA dataset using *CD3, CD8, ESR1, HER2, HLA*, and interferon signaling associated gene expression

To test our hypotheses that high number of mutations would produce more immunogenic mutations and that TILs would be more abundant in those tumors, we analyzed 396 TCGA breast cancer cases according to the PAM50 predictor. *CD3D*, *CD3E*, *CD3G*, *HLA-A*, and *HLA-C* gene expression and the numbers of mutations were higher in *HER2*-enriched and basal-like subtypes than in luminal tumors (Table 5). *CD8B* and *HLA-B* expression were highest in basal-like tumors. *ESR1* gene expression showed a significant negative correlation with *HLA-A* (rho = −0.154, *P* = 0.002) and *CD8B* (rho = −0.315, *P* < 0.001) gene expression (Figure 4).

**Figure 5:**
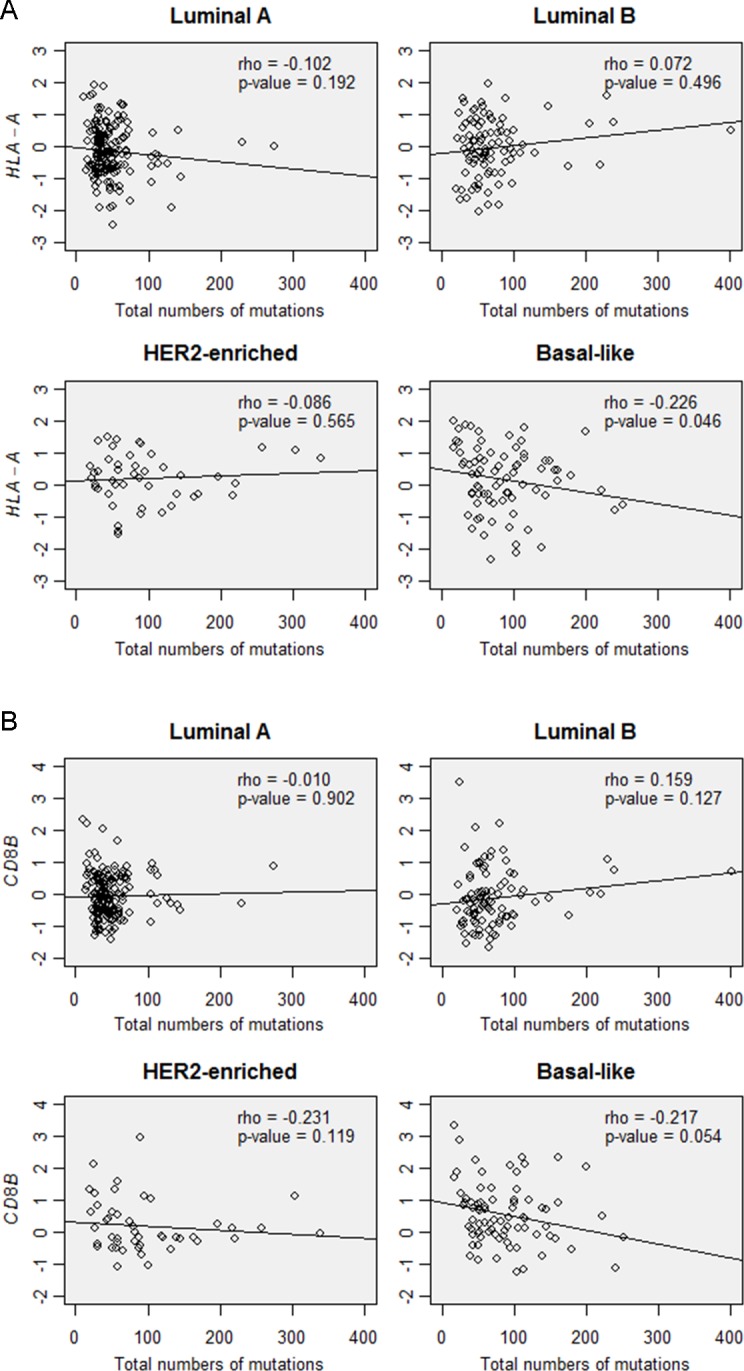
Correlations between the total number of mutations and **A.** *HLA-A* gene expression and **B.** CD8B gene expression in the tumor subtypes of TCGA data.

When all tumors were analyzed, the *CD8B* gene expression level was significantly correlated with the gene expression levels of *CD3D*, *CD3E*, *CD3G*, *HLA-A*, *HLA-B*, and *HLA-C* (rho = 0.448-0.792, *P* < 0.001). However, *CD8B* gene expression was not associated with the total number of mutations (rho = 0.029, *P* = 0.566). *HLA-A* gene expression also showed no correlation with the total number of mutations (rho = −0.026, *P* = 0.603). In subgroup analysis, positive correlation was seen between *CD8B* and *HLA-A* expression in all subtypes (rho > 0.290, *P <* 0.046). However, the total number of mutations was not associated with *CD8B* or *HLA-A* gene expression in each subtype of tumors (Figure 5).

We also analyzed the expression of IFN signaling molecules to assess their expression level in each molecular subtype and their relationship with TILs and *HLA* expression. Expression of the Type 1 and 2 IFN receptor genes (*IFNAR1*, *IFNAR2*, *IFNGR1*, and *IFNGR2*) and IFN inducible gene *MX1* were generally higher in *HER2*-enriced and basal-like subtypes than in luminal tumors (Table 5). Expression of IFN signaling molecules showed a significant positive correlation with *HLA*s, *CD3,* and *CD8* gene expression (Figure 4).

**Table 5:** Comparison of immune-related gene expression and number of mutations according to the molecular subtype in the TCGA data

Variables	Luminal A (*n= 168*)	Luminal B(*n*= 96)	HER2-enriched (*n*= 48)	Basal-like (*n*= 84)	*P*
*ESR1*	0.59 ± 1.07	0.75 ± 0.99	−3.14 ± 2.22	−5.24 ± 1.76	<0.001
*ERBB2*	0.21 ± 0.78	0.13 ± 1.00	1.88 ± 1.39	−0.65 ± 0.89	<0.001
*CD3D*	−0.10 ± 0.96	0.01 ± 1.13	0.44 ± 1.02	0.45 ± 1.25	<0.001
*CD3E*	−0.5 ± 0.36	0.04 ± 0.46	0.24 ± 0.41	0.24 ± 0.48	<0.001
*CD3G*	−0.16 ± 1.24	−0.17 ± 1.38	0.42 ± 1.32	0.34 ± 1.61	0.009
*CD8B*	−0.04 ± 0.68	−0.11 ± 0.89	0.23 ± 0.81	0.60 ± 1.00	<0.001
*HLA-A*	−0.13 ± 0.78	0.01 ± 0.92	0.17 ± 0.81	0.18 ± 1.01	0.012
*HLA-B*	−0.09 ± 0.89	0.03 ± 1.01	0.02 ± 1.01	0.13 ± 1.20	0.288
*HLA-C*	−0.11 ± 0.69	−0.02 ± 0.78	0.09 ± 0.73	0.11 ± 0.86	0.04
*MX1*	−0.18 ± 0.83	0.03 ± 1.05	0.01 ± 0.71	0.07 ± 1.04	0.015
*IFNAR1*	−0.08 ± 0.33	0.06 ± 0.48	−0.31 ± 0.51	0.10 ± 0.56	<0.001
*IFNAR2*	−0.13 ± 0.43	­–0.07 ± 0.58	0.27 ± 0.61	0.79 ± 0.59	<0.001
*IFNGR1*	0.05 ± 0.55	−0.31 ± 0.53	0.20 ± 0.75	0.39 ± 0.69	<0.001
*IFNGR2*	0.04 ± 0.41	−0.17 ± 0.48	0.14 ± 0.44	0.15 ± 0.61	0.001
Total number of insertions and deletions number of frameshift deletion number of frameshift insertion number of in-frame deletion number of in-frame insertion	3.5 ± 3.4 2.0 ± 2.1 0.7 ± 0.8 0.7 ± 1.5 0.2 ± 0.6	4.4 ± 3.4 2.6 ± 2.6 0.7 ±0.8 1.0 ± 1.3 0.1 ± 0.4	3.1 ± 2.3 1.8 ± 1.8 0.7 ± 0.8 0.5 ± 0.7 0.1 ± 0.4	6.3 ± 5.2 3.8 ± 3.3 0.7 ± 1.3 1.5 ± 1.6 0.3 ± 0.5	<0.001
Total number of point mutations number of missense mutation number of nonsense mutation number of nonstop mutation number of RNA mutation number of silent mutation number of splice site mutation	45.1 ± 31.8 28.1 ± 20.1 2.1 ± 2.2 0.1 ± 0.2 2.8 ± 2.7 11.1 ± 8.7 1.1 ± 1.4	68.4 ± 54.3 43.3 ± 35.3 3.4 ± 4.0 0.1 ± 0.3 3.4 ± 2.7 16.9 ± 15.1 1.3 ± 1.4	93.9 ± 73.4 61.6 ± 48.2 5.3 ± 6.2 0.2 ± 0.4 3.4 ± 2.5 21.9 ± 17.4 1.5 ± 1.5	79.6 ± 48.7 52.8 ± 2.9 3.5 ± 3.0 0.1 ± 0.2 4.0 ± 3.4 17.7 ± 1.6 1.6 ± 1.6	<0.001
Total number of mutations	48.6 ± 33.4	72.7 ± 54.7	96.9 ± 73.9	85.9 ± 51.3	<0.001

### Analysis of CCLE data

**Figure 6:**
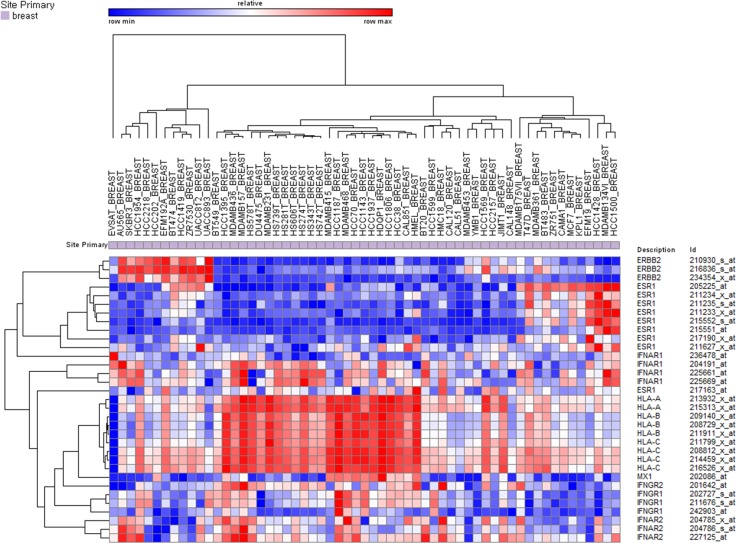
Unsupervised hierarchical clustering of breast cancer cell lines in the CCLE dataset using *ESR1, HER2, HLA,* and interferon signaling associated gene expression

To confirm the negative correlation between *ESR1* and *HLA* gene expression and the positive correlation between expression level of IFN signaling molecules and *HLA* genes, we analyzed CCLE data set. In 59 breast cancer cell lines, *HLA* gene expression was inversely correlated with *ESR1* expression and positively correlated with expression of IFN associated genes (Figure 6).

## DISCUSSION

To the best of our knowledge, this study is the first to show different level of expression of HLA-ABC in each breast cancer subtype and its close relationship with TILs. We wanted to answer the questions why some breast cancers have abundant TILs and how HLA expression of tumor cells and TILs are involved. We could suggest two possible explanations for the close relationship between TILs and HLA expression. In first, the genomic instability of cancer may produce an immunogenic mutation that triggers an influx of lymphocytes to the cancer. The activated TILs could then produce IFNγ, which is a potent HLA inducer that stimulates *HLA* gene transcription [10]. To test this hypothesis, we analyzed the number of mutations and the gene expression of breast cancer from the TCGA data. Even though we found more mutations and higher expression of *CD3*, *CD8*, and *HLA* genes in *HER2*-enriched and basal-like tumors than in luminal tumors, there was no correlation between the total number of mutations and *HLA* or *CD8* gene expression in any molecular subtype.

Alternatively, HLA expression may be increased due to some unknown mechanism and promote presentation of intracellular molecules on the surface of tumor cells. TILs may then be effectively introduced to the tumor microenvironment. We revealed that the expression of the Type 1 and 2 IFN receptor genes (*IFNAR1*, *IFNAR2*, *IFNGR1*, and *IFNGR2*) and IFN inducible gene *MX1* was generally higher in *HER2*-enriced and basal-like subtypes than in luminal tumors and showed a significant positive correlation with *HLA*s, *CD3,* and *CD8* gene expression in TCGA data. Therefore, increased IFN signaling could be suggested as the cause of increased HLA expression and better antigen presentation on the surface of tumor cells. Interestingly, Sistigu et al. [13] recently found type I IFN autocrine and paracrine circuitries on tumor cells and their prognostic and predictive significance in breast cancer. We also confirmed positive correlation between expression of *HLA*s and IFN associated molecules in CCLE data. Since breast cancer cell lines are only composed of epithelial cells, the effect of IFNγ produced by lymphocytes for the induction of *HLA* gene expression can be excluded, which in turn, emphasizes the importance of autonomous IFN signaling in tumor cells. Mostafa et al. [15] reported that estradiol-ERα signaling plays a negative role in IFNγ inducible MHC II expression *via* reducing expression of class II transactivator (CIITA), which increases expression of MHC I and II, in breast cancer cells. Further research to explore the mechanism of increased IFN signaling in breast cancer is needed to improve our understanding about TILs.

We found a negative correlation between ER and HLA-ABC protein expression in our consecutive breast cancer cohort, non-neoplastic breast tissues, and normal luminal cells of cancer patients. Santagata et al. [16] meticulously examined the expression of various markers, including ER, CK5/14/17, and Ki67, and found four mutually exclusive subsets of luminal cells in reduction mammoplasty tissue: ER^+^, CK5/14/17^+^, ER^−^CK5/14/17^−^, and Ki67^+^ cells. In that study, each lobule showed different enrichments of ER^+^, CK5/14/17^+^, and Ki67^+^ cells. We also found various ER expression patterns in each normal tissue sample. For example, we found a lobule with high ER and low HLA-ABC expression and another lobule with low ER and high HLA-ABC expression. We also confirmed a negative correlation between *ESR1* and *HLA* gene expression in TCGA and CCLE data.

From our present findings, we speculate that a low number of TILs in HR^+^ tumors may be associated with a higher expression of ER and a lower expression of HLA. Therefore, immune checkpoint inhibitors, which are increasingly being used and require pre-existing CD8^+^ T cells as a biologic target, may not be so effective in HR^+^ breast cancer [17]. To promote a higher immune response, strategies that can increase expression of the HLA protein may be helpful. Recently, some studies have reported the ability of low-dose chemotherapy and radiation to modify the tumor microenvironment [18, 19]. Chemotherapy can induce inflammatory cytokines that increase T cell influx whereas radiation can also enhance Fas expression and Fas-dependent cytotoxic lymphocyte killing of tumor cells and increase MHC I molecules. In addition, further studies exploring the relationship between ER and HLA expression in breast tissue and the mechanism of TIL enrichment in TNBC are warranted to discover effective ways to improve patients’ immune responses to cancer.

In summary, HLA expression is inversely correlated with ER expression in normal luminal cells and breast cancer and positively correlated with TILs and expression of IFN-associated molecules in breast cancer. Further investigation of the mechanism of how higher HLA expression and TIL influx are related in TNBC may help us find new therapeutic strategies to boost the host immune response.

## MATERIALS AND METHODS

### Patients and tissue specimens

Two sets of breast cancer and one set of non-neoplastic breast tissue samples were used. The first series included 688 consecutive breast cancer patients who underwent surgery for primary breast cancer between 1993 and 1998 at Asan Medical Center, Seoul, Korea, and who had formalin-fixed, paraffin-embedded, tissue samples for analysis. A total of 769 TNBC patients between 2004 and 2010 at Asan Medical Center were included as the second series (Supplementary Data). All patients were preoperatively chemo- and radiotherapy naïve.

For non-neoplastic breast samples, 15 cases of mammary hamartoma and 26 cases of reduction mammoplasty were included. No patients had a history of breast cancer. Clinicopathologic information was obtained from the patients’ medical records and surgical pathologic reports. Exemption from informed consent after de-identification of information was approved by the Institutional Review Board of Asan Medical Center. This study has well followed the REMARK guidelines [20].

### Histological evaluation

The hematoxylin and eosin (H&E)-stained slides were reviewed by two pathologists (H.J.L. and G.G.). Slides were histopathologically analyzed for TILs (defined as the percentage of stroma of invasive carcinoma infiltrated by lymphocytes in 10% increments; if less than 10% of stroma was infiltrated by TILs, 1% or 5% criteria were used; all available full-sections were evaluated),histologic subtype and grade, tumor size, pT stage, pN stage, and lymphovascular invasion [1, 21]. The histologic type was defined based on the 2012 WHO classification criteria, and the histologic grade was assessed using the modified Bloom-Richardson classification [22].

### Cell lines

Human breast cancer cell lines MDA-MB-231, MDA-MB-468, MDA-MB-436, T47D, BT474, BT20, ZR-75-1, MCF-7, and SK-BR-3 from American type culture collection were used as controls for HLA-ABC immunohistochemistry. Cells were grown in RPMI-1640 media with 10% fetal bovine serum and 1% penicillin-streptomycin. Cell lines have been tested and authenticated and verified as mycoplasma free. Formalin-fixed, paraffin-embedded cell blocks were generated.

### Tissue microarray construction and immunohistochemical evaluation

Formalin-fixed, paraffin-embedded, tissue samples were arrayed with a tissue-arraying instrument as previously described [23]. Each sample was arrayed in three 1-mm diameter cores to minimize tissue loss and overcome tumor heterogeneity. Tissue microarray sections were stained with an automatic immunohistochemical staining device (Benchmark XT; Ventana Medical Systems, Tucson, AZ). Antibodies used in this study are summarized in Supplementary Table S3. As controls for HLA-ABC, cell blocks from human breast cancer cell lines were used (Supplementary Data and Supplementary Figure S1).

ER and PR levels were regarded as positive if there was at least 1% positive tumor nuclei staining [24]. In addition, the Allred score, which is the sum of intensity score (0-3) and proportion score (0-5), was calculated for ER and PR [25]. HR^+^ tumors are defined as those with positive ER and/or PR. HER2-overexpressing tumors were defined as those with scores of 3+ by immunohistochemistry or gene amplification by silver in situ hybridization (SISH) [26]. For CK5/6 and epidermal growth factor receptor (EGFR), any positive cytoplasmic and membranous staining was considered as positive [23]. The percentage of cytoplasmic CK5-positive cells was evaluated in normal tissue.

The HLA-ABC intensity was evaluated as a four-value intensity score (0, 1, 2, and 3). The percentage of membranous and/or cytoplasmic expression of HLA-ABC was also evaluated. An “immunoreactive score” was generated as the product of the intensity and the percentage of positive cells. We correlated immunoreactive score of HLA-ABC with ER Allred score. We also categorized HLA-ABC expression in tumor cells as one of three levels as previously described [strongly positive, expression in more than 75% of tumor cells; weakly positive, expression between 25% and 75% of tumor cells; negative, loss of expression in more than 75% of tumor cells] [12].

The immunostained tissue microarray slides for CD8 and CD69 were scanned using a digital microscopy scanner Pannoramic 250 FLASH (3DHISTECH Ltd., Budapest, Hungary). The numbers of CD8^+^ and CD69^+^ cells in tissue microarray cores were counted by the NuclearQuant module of Pannoramic Viewer 1.15.2 (3DHISTECH Ltd).

### TCGA and CCLE data

The TCGA data portal was used to download the breast cancer data [27]. A total of 396 cases of breast cancer were analyzed according to the PAM50 predictor. We downloaded level 3 gene expression data derived from an Agilent custom 244K whole genome microarray and somatic mutation data using exome sequencing from Illumina Genomic Analyzer. Gene expression level was median centered by genes.

Gene expression of 59 breast cancer cell lines using GeneChip Human Genome U133 Plus 2.0 Array included in CCLE was analyzed using GENE-E software, version 3.0.230 [28].

The five analysis sets are shown in Supplementary Table S4 where we have indicated the specific evaluations that were performed for each set of data.

### Statistical analysis

All statistical analyses were performed using SPSS statistical software (version 18; SPSS, Chicago, IL). A Kruskal-Wallis test, chi-square test, linear-by-linear association test, Spearman's correlation, log-rank test, and Cox proportional hazards regression model were used as appropriate. All tests were two-sided and statistical significance was set at 5%.

## SUPPLEMENTARY MATERIAL TABLES AND FIGURE


