# Supplementary material for: Differential expression of major histocompatibility complex class I in subtypes of breast cancer is associated with estrogen receptor and interferon signaling
Source: Oncotarget. 2016 Apr 18;7(21):30119–32. doi: 10.18632/oncotarget.8798 (PMC5058668; doi:10.18632/oncotarget.8798)
Supplement: Supplementary file 1 [file oncotarget-07-30119-s001.pdf]

Differential expression of major histocompatibility complex class I in subtypes of breast cancer is associated with estrogen receptor and interferon signaling

Supplementary Material

Patients and tissue specimens

The first series included 688 consecutive breast cancer patients who underwent surgery for primary breast cancer between 1993 and 1998 at Asan Medical Center, Seoul, Korea. This series had formalin-fixed, paraffin-embedded, tissue samples for analysis. Six cycles of adjuvant methotrexate-based regimen (cyclophosphamide 500 mg/m^2, methotrexate 40 mg/m^2, and 5-fluorouracil 500 mg/m^2) or anthracycline-based regimen (cyclophosphamide 500 mg/m^2, adriamycin 50 mg/m^2, and 5-fluorouracil 500 mg/m^2) were administered in 435 patients out of the 688 patients in the consecutive breast cancer cohort. Fourteen patients received adjuvant chemotherapy with unknown regimens. Radiotherapy and hormone therapy was performed in 26.6% and 73.4% of patients, respectively. The median follow-up duration was 15.9 years.

The validation TNBC cohort consisted of 769 TNBC patients who underwent surgery for primary breast cancer between 2004 and 2010 at Asan Medical Center, Seoul, Korea. This cohort of patients had formalin-fixed, paraffin-embedded, tissue samples for analysis. Of the 769 patients in the validation TNBC cohort, 518 were treated with four cycles of adjuvant anthracycline and cyclophosphamide (AC; adriamycin 60 mg/m^2 and cyclophosphamide 600 mg/m^2). The remaining 250 patients were treated with either four cycles of AC followed by four cycles of paclitaxel (175 mg/m^2) or four cycles of AC followed by four cycles of docetaxel (75 mg/m^2). Most patients treated with AC (95.9%) had no lymph node metastasis, whereas most patients treated with AC and taxane (98.4%) had lymph node metastasis. Radiotherapy was performed in 78.5% of patients. None of the patients received hormone therapy. The median follow-up duration was 6.4 years.

Supplementary Table S1. Comparison of pathologic factors in the validation TNBC cohort according to HLA-ABC expression in tumor cells

Variables	HLA-ABC expression			*P*
	Negative	Weakly positive	Strongly positive	
Histology				<0.001
Invasive carcinoma of no special type	134 (76.6)	101 (75.4)	367 (82.5)	
Adenoid cystic carcinoma	3 (1.7)	1 (0.7)	0 (0.0)	
Carcinoma with apocrine differentiation	12 (6.9)	7 (5.2)	8 (1.8)	
Carcinoma with medullary features	0 (0.0)	0 (0.0)	20 (4.5)	
Invasive micropapillary carcinoma	7 (4.0)	6 (4.5)	11 (2.5)	
Mucinous carcinoma	1 (0.6)	0 (0.0)	0 (0.0)	
Metaplastic carcinoma	14 (8.0)	19 (14.2)	39 (8.8)	
Invasive lobular carcinoma	4 (2.3)	0 (0.0)	0 (0.0)	
Histologic grade				<0.001
1	0 (0.0)	0 (0.0)	1 (0.2)	
2	69 (39.4)	39 (29.1)	83 (18.7)	
3	106 (60.6)	95 (70.9)	361 (81.1)	
pT				0.264
1	68 (38.9)	63 (47.0)	192 (43.1)	
2	97 (55.4)	68 (50.7)	239 (53.7)	
3	10 (5.7)	2 (1.5)	14 (3.1)	
4	0 (0.0)	1 (0.7)	0 (0.0)	
Lymphovascular invasion				0.145
Negative	124 (71.3)	105 (78.4)	340 (77.4)	
Positive	50 (28.7)	29 (21.6)	99 (22.6)	
Lymph node metastasis				0.147
Negative	101 (57.7)	98 (73.1)	293 (65.8)	
Positive	74 (42.3)	36 (26.9)	152 (34.2)	
Radiation therapy				0.002
Negative	54 (30.9)	23 (17.2)	82 (18.4)	
Positive	121 (69.1)	111 (82.8)	363 (81.6)	
Adjuvant systemic therapy				0.103
Negative	0 (0.0)	0 (0.0)	1 (0.2)	
AC	107 (61.1)	101 (75.4)	300 (67.4)	
ACT	68 (38.9)	33 (24.6)	144 (32.4)	
Basal type				<0.001
Negative	85 (48.6)	49 (36.6)	138 (31.0)	
Positive	90 (51.4)	85 (63.4)	307 (69.0)	
Tumor-infiltrating lymphocytes				<0.001
≤10%	74 (42.3)	46 (34.3)	61 (13.7)	
20%–30%	43 (24.6)	39 (29.1)	87 (19.6)	
40%–60%	34 (19.4)	19 (14.2)	105 (23.6)	
>60%	24 (13.7)	30 (22.4)	192 (43.1)	
HLA-ABC intensity in stromal cells				
1	11 (6.4)	1 (0.7)	1 (0.2)	<0.001
2	96 (55.5)	67 (50.0)	89 (20.0)	
3	66 (38.1)	66 (49.3)	355 (79.8)	

AC, anthracycline and cyclophosphamide; ACT, anthracycline, cyclophosphamide, and taxane.

Supplementary Table S2. Survival analyses of clinicopathologic variables that affect clinical outcomes in the validation TNBC cohort

Variables	Univariate			Multivariate		
	Hazard ratio	95% CI	*P* value	Hazard ratio	95% CI	*P* value
Age: ≥50 years vs. <50	0.855	0.595–1.228	0.396	0.796	0.557-1.138	0.211
Histologic grade: 3 vs. 1/2	0.756	0.518–1.102	0.146	1.001	0.680-1.472	0.997
pT: 3/4 vs. 1/2	2.876	1.549–5.339	0.001	1.764	0.911-3.413	0.092
Lymph node metastasis: positive vs. negative	2.543	1.789–3.614	<0.001	2.413	1.696-3.433	<0.001
TILs (per 10%)	0.982	0.975-0.989	<0.001	0.980	0.973-0.988	<0.001
HLA-ABC expression: strongly/weakly positive vs. negative	0.670	0.458–0.981	0.038	0.971	0.657-1.435	0.882

CI, confidence interval; TIL, tumor infiltrating lymphocyte.

Supplementary Table S3. List of antibodies used in this study

Antibody	Dilution	Source
ER	1:200	Leica Biosystems, Newcastle, UK
PR	1:200	Leica Biosystems
HER2	1:8	Ventana Medical Systems, Tucson, AZ
EGFR pharmDx	none	DAKO, Glostrup, Denmark
CK5/6	1:200	DAKO
CK5	1:200	Leica Biosystems
HLA-ABC [EMR 8-5]	1:1600	Abcam, Cambridge, UK
CD8	1:200	DAKO
CD69	1:5	Abcam

Supplementary Table S4. Description of the five analysis sets in terms the specific evaluations performed for each set of data

	Estrogen receptor	MHC I	Tumor-infiltrating lymphocyte	Interferon signaling pathway	Mutation analysis
Consecutive breast cancer cohort	ER IHC	HLA-ABC IHC	H&E	—	—
Validation TNBC cohort	ER IHC	HLA-ABC IHC	H&E, CD3 & CD8 IHC	—	—
Normal luminal cells	ER IHC	HLA-ABC IHC	—	—	—
TCGA data	*ESR1* gene expression	*HLA-A*, *HLA-B*, *HLA-C* gene expression	*CD3D*, *CD3E*, *CD3G*, *CD8B* gene expression	*MX1*, *IFNAR1*, *IFNAR2*, *IFNGR1*, *IFNGR2* gene expression	Numbers and types of mutation
CCLE data	*ESR1* gene expression	*HLA-A, HLA-B, HLA-C* gene expression	—	*MX1*, *IFNAR1*, *IFNAR2*, *IFNGR1*, *IFNGR2* gene expression	—

CCLE, Cancer Cell Line Encyclopedia; H&E, hematoxylin and eosin staining; IHC, immunohistochemistry; TCGA, The Cancer Genome Atlas; TNBC, triple-negative breast cancer.

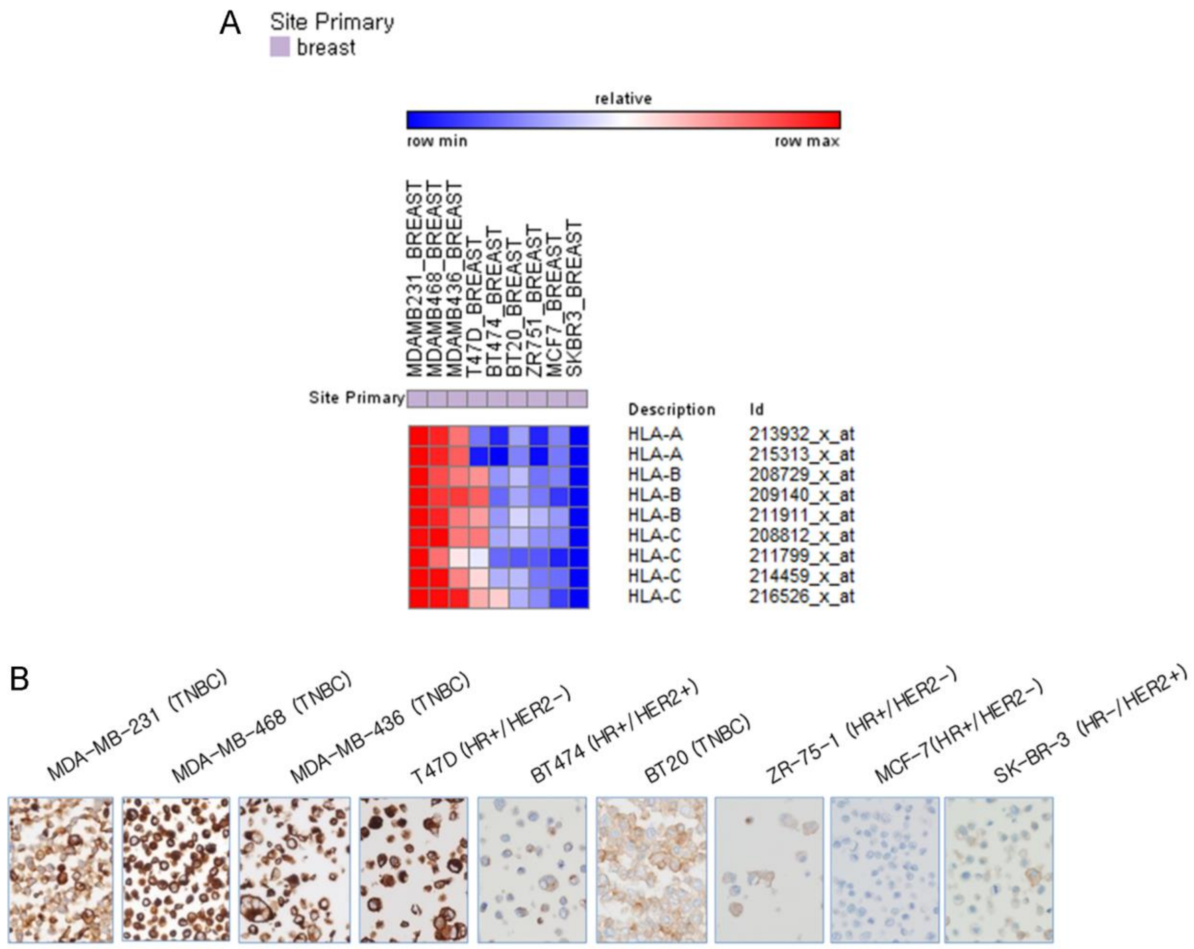

Supplementary Figure S1. *HLA* genes (A) and HLA-ABC protein expression (B) of breast cancer cell lines.
